# Customer’s decision and affective assessment of online product recommendation: A recommendation-product congruity proposition

**DOI:** 10.3389/fpsyg.2022.916520

**Published:** 2022-09-23

**Authors:** Yu Liu, Muhammad Bashir Khan, Muhammad Ashraf, Orangzab, Wareesa Sharif, Jamil Ahmad

**Affiliations:** ^1^Department of Psychology, Guizhou Minzu University, Guiyang, Guizhou, China; ^2^Department of Government and Public Policy, Faculty of Contemporary Studies, National Defence University, Islamabad, Pakistan; ^3^Department of Management Sciences, COMSATS University Islamabad, Vehari, Pakistan; ^4^Department of Artificial Intelligence, The Islamia University of Bahawalpur, Bahawalpur, Pakistan; ^5^Sci-Tech Department, University of Science and Technology of China, Hefei, China

**Keywords:** decision effort, decision quality, enjoyment, system generated recommendation, consumer generated recommendation, cognitive fit theory, schema congruity theory

## Abstract

Online product recommendation (OPR) systems have gained prominence in the context of e-commerce over the past years. Despite the increased research on OPR use, less attention has been paid to examining how decision and affective assessment of the OPR are contingent upon the product type. This study proposes and examines a recommendation-product congruity proposition based on cognitive fit and schema congruity theories. The proposition states that when the content (i.e., a stimulus-based schema) of the OPR [either system-generated recommendation (SGR) or a consumer-generated recommendation (CGR)] matches the brain-stored schema initiated by a particular product (either a search product or an experienced product), then a consumer would use a schema-based information assessment strategy and experience favorable decision and affective assessment of the OPR. This then affects consumers’ intentions to purchase and reuse OPR. The proposition is tested *via a* 2 × 2 between-respondents factorial design of a cross-sectional survey with 482 Amazon customers. The results support the following two matching conditions of the proposition: (1) SGR describing a search product and (2) CGR explaining an experienced product, which might lead customers to perceive lower decision effort, greater decision quality, and higher enjoyment with the OPR that subsequently have a significant impact on their intentions to purchase and reuse OPR. This study expands our understanding of how recommendation-product congruence influences the consumer’s decision and affective assessment behavior and provides practical implications for the identification and presentation of the recommendation type and product type for a better customer decision.

## Introduction

Global B2C e-commerce sale was predicted to rise from $3.53 trillion in 2019 to $6.54 trillion in 2022 (Celement, 2020). It is because of the way e-commerce has been continuously improving due to the shopping ability of the customers and sharing their post-consumption experiences. Online retailers (e.g., Amazon) provide online product recommendations to assist customers in their buying process. They also rely on various types of recommender systems to implement a differential pricing strategy (Zhou et al., 2022). The online product recommendation (hereafter it is called OPR) is considered by academic publications (e.g., Baum and Spann, 2014; Lin, 2014; Asraf et al., 2020; Liao et al., 2021) as important predictors of customer’s product evaluation. The OPR is usually based on system-generated recommendation (hereafter it is called SGR) and consumer-generated recommendation (hereafter it is called CGR) referring to attribute- and experience-based recommendations, respectively (Benlian et al., 2012; Huang et al., 2013).

The system-generated recommendation consists of highly objective information that examines various attributes of a product (e.g., the camera quality of a digital camera) to facilitate customers in deciding types of product to be purchased (Xiao and Benbasat, 2007). The attribute-based recommendation is generated on the basis of customers’ past buying behavior or specified preferences or the preferences of other like-minded customers (Xu et al., 2014). On the contrary, the CGR emphasizes on post-consumption experience consisting of highly subjective information (e.g., the stylish look of a digital camera) (Mudambi and Schuff, 2010).

Online product recommendations have been increasingly available on e-commerce sites and are becoming popular among online customers as they reduce information overload and improve decision quality (Park and Lee, 2009). For example, a consumer survey reveals that 85% of the customers consult CGR for evaluating a product, and 65% claim that CGR influences their purchase decision. Several past studies (e.g., Xiao and Benbasat, 2007; Huang et al., 2013; Ashraf et al., 2020) have also emphasized that the OPR can impact the customers’ beliefs and behavioral reactions in the decision process. Past studies have also separately investigated the effectiveness or the economic impact of SGR (e.g., Oestreicher-Singer and Sundararajan, 2012) and CGR (e.g., Mudambi and Schuff, 2010; Ullal et al., 2021), and their individual impact on various customer beliefs or product choice (e.g., Xu et al., 2014), but the findings about the assessment of the OPR (SGR and CGR) have been mixed.

Huang et al. (2013) claimed that attribute-based recommendation (i.e., SGR) could be more informative than experience-based recommendation (i.e., CGR) because the former emphasizes on evaluating tangible attributes than the intangible attributes of a product. In contrast, Bei et al. (2004) found that compared to attribute-related information (i.e., SGR), experience-related information (i.e., CGR) is more valuable to evaluate a product. For instance, Ashraf et al. (2019) found that users of SGR express significantly higher perceived enjoyment than users of CGR, while users of CGR (compared to SGR) express higher trust beliefs. The mixed findings might be due to the different types of products described in the contents of OPR because the evaluation of different products requires different types of information (Mudambi and Schuff, 2010). Relatively less attention has been paid to understanding the way different types of recommendations for different products are evaluated (Whang and Im, 2021). Therefore, an interaction impact between the OPR type (SGR vs. CGR) and product type (search vs. experience) on customers’ evaluation beliefs may explain how SGR and CGR are evaluated in e-commerce transactions.

In order to reconcile the diverse findings, we speculate that the different products (i.e., search and experience) described by the OPR influence customer’s decisions and the affective assessment of OPR. Based on the cognitive fit theory (CFT) (Vessey and Galletta, 1991), a customer’s decision and affective assessment of OPR are enhanced by the fit between incoming information (i.e., SGR or CGR) and the particular task (i.e., buying the search product or an experienced product). It subsequently leads the OPR users to form a consistent mental representation that is explained by schema congruity theory (SCT) (Meyers-Levy and Tybout, 1989). A customer’s intention for searching information (SGR and CGR) on different products may activate brain-stored schema (i.e., particular information in the brain). A fit between the schema of incoming information and the brain-stored schema allows the customers to formulate a consistent representation. By nature, there are two types of products: search and experience products (Mudambi and Schuff, 2010). Customers’ information searching on experience product and search product would activate an analytic brain-stored schema and holistic brain-stored schema, respectively (Huang et al., 2013). Therefore, we examine how different types of products would influence customers’ decisions and the affective assessment of SGR and CGR.

In line with the CFT and SCT, this study formulates a recommendation-product congruity proposition: when the content (i.e., a stimulus-based schema) of the OPR (i.e., SGR vs. CGR) matches the brain-stored schema initiated by the focal product type (search vs. experience), then OPR users would use a schema-based information assessment strategy and experience a favorable decision (represented by perceived decision effort and decision quality) and affective (represented by perceived enjoyment) evaluation of the OPR because both the schemas are consistent in processing information which in turn influences their intentions to purchase and reuse OPR.

This study particularly contributes to the existing literature in the following four ways. First, this study reconciles with the prior findings by arguing that product type is required to be considered for decision and affective assessment of OPR. Second, we observed that the CFT theorizes on the matching condition between the focal product type and OPR type which would facilitate the customer’s decision and affective assessment of the OPR. Third, this matching condition could be significantly refined by incorporating the CFT with the SCT, which explains how brain-stored schemas guide the customer’s behavior for subsequent acquisition and processing of the product-related information in order to create consistent mental representation. Such an explicit integration of CFT and SCT clarifies the theoretical argument on how a mental fit occurs between an incoming recommendation and shopping task in a customer’s evaluation of OPR, thereby expanding the CFT. Fourth, by considering perceptual measures of customers’ decisions and affective beliefs of OPR evaluation, this study reconciles with the different past views on the impact of matching the conditions on the customers’ decision and affective assessment of the OPR.

## Theoretical background

### Recommendation type: System-generated recommendation vs. consumer-generated recommendation

Recommendations are usually categorized into four types based on their stemming sources (Senecal and Nantel, 2004; Benlian et al., 2012): First, the personal source providing personalized information; second, the personal source providing non-personalized information (i.e., CGR); third, the impersonal source providing personalized information (i.e., SGR); and fourth, the impersonal source providing non-personalized information. In this study, we consider second (i.e., CGR) and third (i.e., SGR) types of OPR as both compensate for the absence of product quality inspection in an online environment (Baum and Spann, 2014; Lin, 2014; Liao et al., 2021). They (i.e., SGR and CSGR) are extensively deployed on e-commerce platforms and used by online customers for making purchase decisions. Moreover, they exhibit distinct characteristics, having a differential impact on customers’ beliefs and behavior (Ashraf et al., 2019). Additionally, it would be interesting to explore how they influence customers’ beliefs and behavior.

Furthermore, SGR and CGR can be differentiated based on their various characteristics. First, the contents of SGR and CGR are generated by a recommender system and past consumers, respectively. The CGR depends upon consumers’ original and first-hand experience with the product usage, whereas the SGR relies on the recommender system which interferes in the recommendation generation process. Although the CGR is not generated by an information system, it is mediated by the system to generate recommendations (Benlian et al., 2012; Ashraf et al., 2019). Second, based on the content or collaborative-based technique, the recommender system automatically generates recommendations (i.e., SGR) by statistically processing the data of consumers’ buying behavior or affinity group (Xu et al., 2014), whereas the CGR is based on data points extracted from consumers’ first-hand usage experiences with the product (Mudambi and Schuff, 2010). Third, the SGR is presented in a standardized format consisting of text, pictures, and multimedia files, whereas the CGR mostly consists of a textual paragraph in a non-standardized and non-consistent format (Benlian et al., 2012). Fourth, online sellers have full control over the structural format of the SGR presentation compared to the CGR presentation. Fifth, in general, the SGR is considered more personalized and objective, whereas the CGR is perceived to be impersonal and subjective (Lin, 2014; Xu et al., 2014). Past studies have relatively underscored the significance of investigating the influence of recommendation content (SGR/CGR) on customers’ decisions and affective assessment of OPR. As discussed above, the distinct characteristics of SGR and CGR have a differential influence on the customer’s beliefs on OPR evaluation.

A critical but typically unnoticed aspect of OPR is the way the OPR assists customers in purchase decisions (Ullal et al., 2021). The OPR (SGR/CGR) can express a product evaluation based on either product attributes or consumers’ post-consumption experiences (Ashraf et al., 2019). For instance, for a smartphone evaluation, the OPR content might consist of attribute-based information related to the processor, camera pixel, and the network quality of the phone. Such a recommendation is considered an SGR that focuses on the objective evaluation of the product. Contrarily, the OPR content might contain post consumption-based evaluation of the smartphone. Such a recommendation is referred to as CGR that focuses on the subjective evaluation of the phone. Past studies have reported mixed findings on the influence of the OPR (SGR/CGR) use on consumers’ OPR evaluation beliefs in e-commerce transactions. For example, Benlian et al. (2012) investigated the differential effect of SGR and CGR and reported that users of SGR (compared to CGR) express greater usefulness and ease of use, while the users of CGR (compared to SGR) express greater effectiveness and trusting beliefs, resulting in different effect mechanisms in e-commerce transactions.

Whereas Ashraf et al. (2019) found that CGR (compared to SGR) is more effective for decision quality and SGR (compared to CGR) significantly reduces the decision effort. Similarly, Bei et al. (2004) found that experiential information (i.e., CGR) is more useful compared to attribute-based information (i.e., SGR). These mixed findings might be due to the different products described in the OPR content, which causes users’ perception of OPR evaluation contingent upon the nature of the products (Huang et al., 2009; Mudambi and Schuff, 2010).

### Product type: Search vs. experience

From the perspective of pre-purchase performance veracity, there could be two types of products: search and experience (Willemsen et al., 2011). The search product can be defined as a product that can be evaluated based on its attributes of objective nature and does not require experiential evaluation. Whereas the experience product is a product that cannot be evaluated based on technical parameters of product attributes and requires subjective evaluation of the product attributes which is a matter of personal tastes. Search products, such as laptops (Huang et al., 2013) or electronics (Willemsen et al., 2011), are characterized by functional and concrete attributes which can be correctly examined before product use. Experience products, such as shoes (Huang et al., 2013) or recreational services (Willemsen et al., 2011) are characterized by intangible attributes which cannot be accurately examined before product use and thus requires post-consumption information for greater scrutiny of the product. Experience product is difficult to be evaluated and compared based on the objective information of their attributes; it requires experience-related information based on one’s senses to examine the product quality (Weathers et al., 2007; Mudambi and Schuff, 2010). Therefore, understanding the differing information needs for evaluating the different products (search vs. experience) can inform us about the effectiveness of the OPR type (SGR vs. CGR) in e-commerce transactions.

Several past studies (e.g., Mudambi and Schuff, 2010; Ashraf et al., 2020) have examined the influence of product type on information processing, the way SGR and CGR are evaluated for the distinct nature of products, and exploring their interactive impact on customer’s decision (decision effort & decision quality); the affective (perceived enjoyment) assessment of OPR is largely ignored in the past literature. The interaction effect of OPR type and product type can be explained based on CFT and SCT.

### Cognitive fit theory

The cognitive fit theory proposes that the correspondence between information presentation and task leads to better task performance. The CFT had been applied in the context of decision making by describing how a fit between the problem representation (e.g., the provision of recommendation content) and problem-solving task (e.g., product evaluation) results in favorable behavior due to the development of consistent mental representation. Whereas consistent mental representation refers to the way a person conceives a problem (Vessey and Galletta, 1991). Several prior studies have empirically tested the CFT in various research domains (e.g., Choi and Gil-Garcia, 2022; Jiang et al., 2021). For instance, Chandra and Krovi (1999) examined how the fit between external information and internal representation develops the congruence of cognitive representation in retrieving the information. Hong et al. (2004) demonstrated that visual aid facilitates the match between information representation and online shopping tasks. Huang et al. (2013) validated the CFT in the context of product reviews so that attribute-based reviews describing search products and experience-based reviews explaining experience products lead customers to perceive greater helpfulness and lower cognitive effort in comprehending the reviews. Hence, the common theme underlying these studies is that if both problem representation and problem-solving tasks emphasize the same information, then a cognitive fit is attained which subsequently leads to the development of a consistent mental representation.

Although the CFT predicts the fit between the problem representation and the problem-solving task for deriving a solution to the problem, it fails to explain or expose the underlying cognitive mechanisms for developing the consistent mental representation (Huang et al., 2013). However, this study is also based on the SCT which complements the CFT in providing an explanation for the underlying matching condition.

### Schema congruity theory

The schema congruity theory was developed to examine how cognitive schema affects the processing of incoming new information in order to formulate consistent mental representation (Meyers-Levy and Tybout, 1989). The processing of new information is influenced by two types of schemas: brain-stored schema and stimulus-based schema (Meyers-Levy and Tybout, 1989). Brain-stored schema refers to a systematized form of beliefs, affects, and expectations that guide thought, perception, and action on the basis of the existing knowledge stored in the brain (Huang et al., 2013; Lee and Kim, 2022). Whereas stimulus-based schema refers to organizing and presenting the incoming information that is external to the brain (Huang et al., 2013). The SCT posits that a customer’s decision task to evaluate the product leads to activating the brain-stored schema. Brain-stored schema for a specific product can be formulated based on the basic impression, understanding, or post-consumption experience of the product (Huang et al., 2013). Even when a customer confronts an unfamiliar product, he/she forms a basic schema combining the product information with the related schemas (Huang et al., 2013). For example, when a digital camera was first launched in the market, customers formulated its basic schema in the brain by conceiving the digital camera as good quality with a higher mega pixel.

Brain-stored schema guides customers’ behavior for subsequent acquisition and processing of information related to a product. The customer’s response to the incoming information represented by the schema relies on the congruity of the schema with the existing brain-stored schema (Lou et al., 2021). The customer follows a schema-based information processing strategy when there is a congruity between the stimulus-based-schema of incoming new information and the existing brain-stored schema (Huang et al., 2013). Consequently, it leads to formulating a consistent mental representation which helps process the information with less cognitive effort. For instance, the brain-stored schema of evaluating a smart cell phone renders a judgment based on its key features, such as the battery, screen, and memory size. When this incoming information is closely matched with the existing related schema, then schema congruity occurs, which helps the customer to process the information for evaluating the product with minimal cognitive elaboration. In contrast, if a mismatch between stimulus-based-schema and brain-stored schema occurs, then schema incongruity leads the customers to adopt a piecemeal strategy of information processing and exerts greater effort in integrating the two schemas (Huang et al., 2013).

Since the OPR is presented in two forms of SGR and CGR providing different information for product evaluation, the SGR provides an objective evaluation of the product by describing its attributes, such as display quality, memory capacity, and the weight of a mobile phone. The CGR contains a subjective evaluation of the product, such as the “stylish design of a mobile phone.” According to the SCT, a customer’s search for a focal product type activates his or her brain-stored schema. For instance, when a customer intends to buy a search product, then he has the expectation to search objective attribute-related information or when a customer wants to buy an experienced product, then he expects to search experience-related information from the subjective perspective of product evaluation (Wang and Wei, 2006; Weathers et al., 2007). Depending on the customers’ intention to evaluate a search- or an experience-product, the brain-stored schema representation of search attribute-related or experience-based related information is initiated and established, respectively (Huang et al., 2013; Lee and Kim, 2022). Therefore, it can be argued that a customer uses a schema-based information assessment strategy when the incoming recommendation type (i.e., stimuli-based-schema of SGR and CGR) matches the brain-stored schema (i.e., the stored structure of SGR and CGR) activated by the product type (search vs. experience). Consequently, both schemas emphasize the same information structure that leads to developing consistent mental representation which may influence customer’s decision and affective assessment of OPR.

## Research model and hypotheses

The main theme of this study is to study the impact of OPR (SGR vs. CGR) on a customer’s decision and the affective assessment of the OPR which is contingent upon the nature of the product.

**FIGURE 1:**
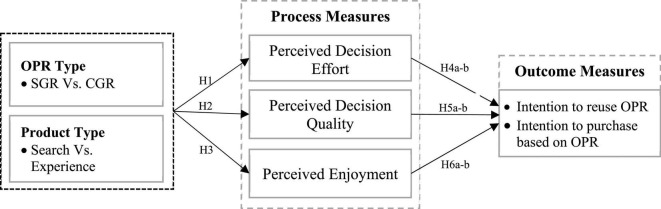
Research model.

Particularly, a fit between the OPR type (SGR and CGR) and product type (search and experience) would produce a favorable customer response to the recommended product. Based on CFT and SGT, it is hypothesized that for a buying task of a search product, the OPR should be objective- and attribute-based (i.e., SGR) because the brain-stored schema favors the information that is based on the technical attributes of the product. For a buying task that involves an experienced product, the OPR should be subjective- and experience-based (i.e., CGR) because the brain-stored schema emphasizes the subjective information that is based on the post consumption experience of the product. However, this study proposes a recommendation-product congruity proposition (Figure 1).

When the content (i.e., a stimulus-based schema) of an online product recommendation (i.e., SGR or CGR) matches the brain-stored schema initiated by a particular product type (search or experience), then the customer would use a schema-based information assessment strategy and experience favorable decision and affective assessment of OPR, because both schemas emphasize the same information structure that results in a consistent mental representation which in turn affects customer’s intentions to purchase and reuse OPR.

Decision assessment of OPR is determined by perceived decision effort and perceived decision quality. Decision effort refers to the extent to which the cognitive effort exerted by a customer in processing the product information for arriving at a purchase decision (Xu et al., 2014). In this study, the decision effort is a perceptual measurement of the amount of time and cognitive resources exerted in evaluating the product based on the OPR content. Whereas perceived decision quality based on OPR is also the perceptual measure of decision confidence and effectiveness. It refers to the extent to which a customer has bought the recommended product that fits his need or taste (Xu et al., 2014). Additionally, the affective assessment of OPR is determined by customers’ perceptual measurement of enjoyment which refers to the extent to which OPR usage is perceived to be enjoyable in its own right apart from any performance consequences which may be expected (Xu et al., 2014). This study also includes two outcome measures, purchase intention and OPR reuse intention, as dependent variables, because the impact of OPR on customers’ buying decisions or product judgment is effective only when customers intend to purchase the recommended product and to reuse the OPR whenever needed to buy a product in the future (Franke et al., 2004).

### Decision assessment of online product recommendation: Decision effort and decision quality

Based on SCT, a particular product buying task could activate varying brain-stored schemas. Particularly, when a customer intends to buy a search product (e.g., laptop), then he expects to seek attribute-related information (i.e., SGR) prior to receiving a recommendation (Weathers et al., 2007; Benlian et al., 2012). However, a brain-stored schema for searching objective and attribute-related information is developed (Meyers-Levy and Tybout, 1989). Similarly, for the shopping task of an experienced product (e.g., shoes), the customer tends to search for experience-related information about the product (Huang et al., 2013).

The system-generated recommendation provides a concise amount of objective and personalized information about product attributes which would reduce the decision effort for evaluating a search product than an experienced product because a search product can be evaluated based on the objective nature of its attributes. The decision of buying search products is typically associated with fact-gathering which is impersonal, objective, and outcome-oriented in nature which is related to SGR (Ashraf et al., 2019). In contrast, the customer’s decision of buying an experienced product based on SGR requires a greater cognitive effort, because the evaluation of the experience product is contingent upon subjective explanation which is a matter of personal taste (Huang et al., 2013). Thus, the evaluation of experience products is associated with comparative explanatory information of product attributes which is more personal, subjective, and process oriented in nature (Schlosser, 2003), which is depicted in CGR.

Integrating the following observation with the SCT and CFT, we propose that when the incoming OPR content (i.e., the stimulus-based schema) matches the brain-stored schema initiated by a particular product (search or experience), then the customer uses a schema-based information-processing strategy to comprehend the product information because both schemas share a similar information structure. In the presence of such an information-processing strategy, a consistent mental representation is developed which results in exerting a lower cognitive effort due to the greater ease of OPR comprehension. Subsequently, the customer finds such OPR easier to process, thus saving time and minimizing cognitive effort. On the contrary, when there is a misfit between the OPR content and the brain-stored schema activated by a particular product, then the customer has to adopt a piecemeal-based information processing strategy that requires him to balance the two schemas and express his preference (Huang et al., 2013). Consequently, an inconsistent mental representation is developed under sthe mismatched circumstance. Therefore, the customer has to spend extra time and exert more cognitive effort in order to address the misfit (Meyers-Levy and Tybout, 1989). Hence, we propose the following hypotheses based on the above discussion:

H1: Perceived decision effort is lower when the OPR type matches the product type (i.e., *SGR matches the search product and CGR matches the experience product*).

Past studies (e.g., Häubl and Trifts, 2000; Ashraf et al., 2019) described that the SGR helps customers to choose the product from products available on e-commerce Websites. The SGR is more effective in improving the customer’s ability to evaluate the search product than the experience product. It is because the evaluation of the search product needs attribute-based information presented in SGR than in CGR. However, users of SGR can examine attributes and monetary values of search products in a more effective way which leads to making better buying decisions. Meanwhile, the SGR is less effective in examining the experience products as they require subjective information which is a matter of personal taste which is presented in CGR than in SGR. As opposed to SGR, the CGR provides a post-consumption experience from the subjective perception of the product evaluation, customers’ perception of product quality after consumption. Consequently, the CGR (compared to SGR) enhances greater decision quality for buying experience products (compared to the search product).

Relating these arguments with the theorization of the CFT and the SCT, it can be observed that a consistent mental representation would be developed when there is a fit between the stimulus-based schema (represented by OPR) and the brain-based schema (represented by product type). Such a consistent mental representation developed based on the match between the OPR type and product type makes the OPR cognitively useful to increase decision quality. However, it is speculated that whenever there is a fit between the OPR type and the product type, such as SGR matches a search product and CGR matches an experienced product, the customer perceives a greater decision quality with the OPR. Concisely, we posit the following hypothesis:

H2: Perceived decision quality is greater when the OPR type matches the product type (i.e., *SGR matches the search product and CGR matches the experience product*).

### Affective assessment of online product recommendation: Perceived enjoyment

Consumer-generated recommendation consists of post-consumption experiences in the form of narratives and stories with different examples having the ability in creating vicarious experiences (Cheong and Morrison, 2008). In the context of persuasion research, the messages or narratives are more emotional, interesting, and persuasive than statistical information (O’keefe, 2002). Information without examples might be perceived as unemotional, vague, and impersonal (Benlian et al., 2012). Consistent with this argument, the CGR containing experiential narratives would be more persuasive and emotional for understanding the underlying reasoning process of experience product acquisition than search product acquisition (Benlian et al., 2012). However, the CGR is more likely to stimulate higher perceived enjoyment for fulfilling the information needs for evaluating the experience product than the search product. The CGR (opposed to SGR) containing subjective and emotive text would result in greater customer enjoyment in the process of experience product evaluation.

In contrast to the experience product, search products do not require in-depth and subjective information, and they can be evaluated based on objective and concise information on technical parameters of product attributes presented in the SGR (compared to CGR). Furthermore, the SGR contains task- and mood-relevant cues (e.g., security seals, attribute quality, and color) that provide greater enjoyment for shopping search products (Parboteeah et al., 2009; Xu et al., 2014). In line with the CFT and the SCT, it is argued that a consistent mental representation might be developed based on the match between the OPR type (SGR vs. CGR) and product type (search vs. experience), making the OPR cognitively enjoyable. However, it is conjectured that whenever there is a fit between the OPR type and the product type, such as SGR matches a search product and CGR matches an experienced product, the customer perceives greater enjoyment with the OPR:

H3: Perceived enjoyment is higher when the OPR type matches the product type (i.e., *SGR matches the search product and CGR matches the experience product*).

### Decision outcomes: Intentions to reuse online product recommendation and purchase

According to the effort-accuracy model (Payne et al., 1993), a typical decision maker often faces two objectives: to minimize decision effort and to maximize accuracy (decision quality). Payne et al. (1993) argued that these objectives are often in conflict because more effort is usually required to increase decision quality. As a recommender system performs resource-intensive information processing jobs of screening, narrowing, and sorting available products, customers can free up some of their processing capacity in evaluating the recommended alternatives and subsequently making effective buying decisions (Häubl and Trifts, 2000; Zhou et al., 2022). Moreover, past information system studies found that users’ beliefs in the decision-making process have a direct impact on their behavioral intention (Häubl and Trifts, 2000; Hostler et al., 2005; Wang and Benbasat, 2009; Xu et al., 2014).

In line with the effort-accuracy model, if customers perceive OPR as an effective decision strategy that reduces decision effort, then they would be more intended to continue using OPR for subsequent buying (Wang and Benbasat, 2009; Xu et al., 2014). On the other hand, if the OPR requires extra cognitive effort for making buying decisions, all other things being equal, the customers would be more likely to rely on their own abilities rather than using OPR. A similar analogy can be applied to the likelihood to purchase the recommended product (Xu et al., 2014). However, we argue that lower decision effort of OPR use would increase the likelihood to reuse OPR and purchase the product recommended by OPR due to their positive spillover effect.

H4a: Perceived decision effort negatively influences customer’s intention to reuse OPR.

H4b: Perceived decision effort negatively influences customer’s intention to purchase based on OPR.

If customers perceive OPR as a decision strategy that assists them in improving their decision quality, then they would be most likely to buy the product and continue using OPR for future purchases (Häubl and Trifts, 2000; Hostler et al., 2005; Wang and Benbasat, 2009; Xu et al., 2014). Conversely, if customers perceive that OPR does not help in improving the decision quality, then they would be more likely to avoid buying the product and discontinue using OPR in the future, all other things being equal, then they would prefer to rely on their own capabilities rather than relying on OPR and also not intend to buy the product (Xu et al., 2014). However, we propose the following hypotheses:

H5a: Perceived decision quality positively influences customer’s intention to reuse OPR.

H5b: Perceived decision quality positively influences customer’s intention to purchase based on OPR.

In addition to the utilitarian value, this study also captures the hedonic value of a customers’ OPR usage through their perceived enjoyment with the OPR. Perceived enjoyment has been used to capture individuals’ emotional states and is considered an important affective belief influencing users’ intention to reuse the recommender system (Kamis et al., 2008; Xu et al., 2014). Prior studies have found that perceived enjoyment had a positive impact on the customers’ likelihood of returning to a website (Koufaris, 2002), mobile app reuse intention (Thong et al., 2006), and e-loyalty (Cyr et al., 2009). Moreover, prior information system studies have also found that users’ perceived enjoyment of the recommendation system has a direct impact on their behavioral intention (Thong et al., 2006; Lee, 2010; Xu et al., 2014). In line with the prior findings, it is posited that the customers’ intentions to reuse OPR and purchase would also be influenced by their perception of enjoyment with the OPR.

H6a: Perceived enjoyment positively influences customer’s intention to reuse OPR.

H6b: Perceived enjoyment positively influences customer’s intention to purchase based on OPR.

## Research methodology

### Construct measurements and data collection

This study employed a quantitative method to test the hypotheses developed based on the research model shown in Figure 1. Data were collected *via* an online survey conducted with the real users of OPR on the Amazon site. We consider Amazon customers as a target population because Amazon is the most popular e-marketplace where the provision for SGR and CGR is a prominent example for providing recommendations to Amazon customers. Moreover, Amazon provides a list of verified customers who have exposure to the OPR (SGR or CGR) during their buying decision.

A survey questionnaire consisting of study measures and demographic characteristics was developed and distributed among Amazon customers using the SurveyMonkey platform. The study measures were adopted from prior studies but validated through an expert panel, a pre-testing, and a pilot test. Measures for perceived decision effort were adopted from the experimental study by Xu et al. (2014). First, two items of the perceived decision quality were adopted from Xu et al. (2014) and the remaining three items from an expert panel, whereas the measures of OPR reuse and purchase intentions were adopted from Ashraf et al. (2019). The construct measures are shown in Table 1.

**TABLE 1:** Construct measurement.

Construct and measurement	Source
**Intention to reuse OPR-** *Consumer’s intention to continue using the similar type of OPR whenever he or she needed to buy a product in the future.* If you needed to buy a product in the future, how likely is it that you would IRU1. Intend to continue using the similar type of OPR in the future? IRU2. Predict your use of the similar type of OPR to continue in the future? IRU3. Plan to continue using the similar type of OPR in the future? IRU4. Continue to pay attention to the similar type of OPR?	Ashraf et al. (2019)
**Intention to purchase** ……. If you actually had the money, how likely is it that you would buy the product recommended on Amazon Web site?	Ashraf et al. (2019)
**Perceived decision effort-** *The extent to which cognitive effort exerted by a customer in processing product information in order to arrive at purchase decision.* PDE1. The product selection task that I went through using OPR was frustrating. PDE2. The product selection task that I went through using OPR was complex. PDE3. The product selection task that I went through using OPR required a lot of effort. PDE4. The product selection task that I went through using OPR took much time.	Xu et al. (2014)
**Perceived decision quality-** *The extent to which customer has bought the recommended product fit his need or taste.* The product chosen from alternatives recommended by OPR, it PDQ1. Suited my preference. PDQ2. Best matched my need.	Xu et al. (2014)
PDQ3. Best choice to buy. PDQ4. Helped me to avoid poor choice. PDQ5. Helped me to make best decision possible.	Expert panel
**Perceived enjoyment-** *The extent to which OPR usage is perceived to be enjoyable in its own right apart from any performance consequences which may be expected.* ENJ1. Using the OPR for online buying was enjoyable. ENJ2. Using the OPR for online buying was pleasurable. ENJ3. Using the OPR for online buying was pleasant.	Thong et al. (2006)
ENJ4. Using the OPR for online buying was entertaining. ENJ5. Using the OPR for online buying was fun.	Expert panel

Responses to the questionnaire were measured on a 5-point Likert scale with endpoints; “*1 for strongly disagree*” and “*5 for strongly agree*,” except for purchase intention and OPR reuse intention which were measured on a 5-point scale type, anchored from “*1 for very unlikely*” to “*5 for very likely.*” A screening question, “*whether the respondent had used OPR (SGR or CGR) over the last six months to at least one product on Amazon Site*,” was also asked to identify the real users of OPR. However, only real responses were used in the data analysis.

### Demographic descriptive

We collected 482 valid responses from OPR users which were subsequently used for examining the interaction effect of SGR and CGR in conjunction with search and experience products on customers’ decisions and affective beliefs. Out of 482 responses, 239 were for SGR use and 243 were for CGR use.

Demographic results reveal that 51.5% are male, 57.9% are married, 39.6% have a bachelor’s degree, and 24.7% are self-employed. Furthermore, 32.4% of the respondents are from the USA and the remaining are from 12 different countries. On average, respondents have been using the Internet for more than 6 years and buying online for over 4–5 years. Furthermore, the respondents have familiarity with Amazon and OPR, and they also frequently visit the Amazon site. Complete detail of the demographic descriptive data is presented in Table 2.

### Common method bias analysis

Since the data were collected through a cross-sectional survey, we examined the common method bias (CMB) by employing Harman’s one-factor test (Podsakoff et al., 2003) and correlation test (Bagozzi et al., 1991; Pavlou et al., 2006). SPSS (version 20) is used to conduct Harman’s one-factor test. In this test, all construct measures are entered together and constrained to generate a single factor for examining the variance. The test result shows that a single factor accounted for 35% of variance is less than 50% of rules of thumb, indicating that the CMB is not a problem in this study. Moreover, we also examined the results of correlation among the variables to determine whether a high correlation exists among variables or not. A high correlation of more than 0.9 indicates that the CMB exists in the study (Bagozzi et al., 1991). As shown in Table 7, none of the correlations is greater than 0.8, indicating that the CMB is not a concern in this study.

**TABLE 2:** Descriptive statistics.

Variable	Frequency (%)
**Age group**	
Less than 20 years	6 (1.2)
20–25 years	39 (8.1)
26–35 years	89 (18.5)
36–45 years	114 (23.7)
46–55 years	109 (22.6)
More than 55 years	125 (25.9)
**Education**	
Certificate	67 (13.9)
Diploma	79 (16.4)
Bachelor’s Degree	191 (39.6)
Master’s Degree	118 (24.4)
Doctorate	27 (5.6)
**Marital status**	
Single	132 (27.4)
Married	279 (57.9)
Living with partner	14 (2.9)
Divorced	32 (6.6)
Widowed	17 (3.5)
Missing values	8 (1.7)
**Occupation**	
Government employed	76 (16.4)
Private employed	110 (22.8)
Self-employed	119 (24.7)
Unemployed	38 (7.9)
Student	49 (10.2)
Retire	87 (18.1)
Missing values	3 (0.6)
**Gender**	
Female	234 (48.5)
Male	248 (51.5)
**Income**	
Under 1,000 USD	34 (5.1)
1,000–2,000 USD	33 (6.8)
2,001–3,000 USD	43 (8.9)
3,001–4,000 USD	67 (13.9)
4,001–5,000 USD	58 (12)
Over 5,000 USD	82 (17)
No income	26 (5.1)
Don’*t* want to disclose	133 (27.6)
Missing values	6 (1.2)
**Geographical location**	
USA	156 (32.4)
UK	81 (16.8)
France	42 (8.6)
Germany	39 (8.7)
Italy	37 (7.7)
Canada	35 (6.6)
Australia	27 (5.6)
Spain	18 (3.7)
India	15 (3.1)
China	9 (1.8)
Brazil	7 (1.5)
Ireland	5 (1.0)
Japan	3 (0.6)
Missing values	8 (1.7)
**Mean (standard deviation)**	
**Internet usage and online buying experience**	
Internet usage experience*	6.83 (0.368)
Online buying experience**	4.75 (1.064)
**Familiarity with Amazon and OPR** (Five-point Likert scale)***	
Familiar with Amazon	4.51 (0.471)
Visit Amazon regularly	4.28 (0.724)
Familiarity with OPR	4.13 (0.691)

*Anchored at 1 = “1–2 years” and 7 = “more than 7 years.”

**Anchored at 1 = “Less than 1 year” and 6 = “more than 5 years.”

***Anchored at 1 = “strongly disagree” and 5 = “strongly agree.”

**TABLE 3:** Respondents’ ability to judge the performance of products before and after purchase.

Products	Before purchase		After purchase		Difference mean	Product type
	Mean	SD	Mean	SD		
Laptop	4.86	1.37	6.52	0.64	1.66	Search
Cell phone	4.75	1.34	6.75	0.63	2.00	Search
Digital camera	4.85	1.36	6.81	0.63	1.96	Search
Home electronics	4.90	1.33	6.49	0.62	1.59	Search
Photographic equipment	4.49	0.58	6.45	0.58	1.96	Search
Motorcycle parts	4.81	0.50	6.79	0.50	1.98	Search
Toys	4.75	1.15	6.58	0.58	1.83	Search
Kitchen utensils	4.61	0.71	5.64	0.71	1.03	Search
DVD player	4.48	0.71	6.25	0.00	1.77	Search
Printer	4.53	0.71	6.56	0.71	2.03	Search
Electronic accessories	4.71	0.58	6.74	0.58	2.03	Search
Network equipment	4.36	1.00	6.15	0.42	1.79	Search
Eyeglasses	6.85	0.42	7.00	0.00	0.15	Search
Software	3.69	1.35	6.25	0.77	2.56	Experience
Books/magazine	3.68	1.37	6.43	0.56	2.75	Experience
Movies/music CDs	3.47	1.34	6.49	0.76	3.02	Experience
Clothing	3.61	1.40	6.61	0.78	3.00	Experience
Shoes	3.74	1.37	6.45	0.76	2.71	Experience
Perfume	3.63	1.37	6.27	0.78	2.64	Experience
Cosmetics	2.49	1.51	6.53	0.79	4.04	Experience
Cleaning products	2.43	0.41	6.18	0.23	3.75	Experience
Pet supplies	3.57	0.71	6.32	0.71	2.75	Experience
Watch	3.69	0.65	6.43	0.71	2.74	Experience
Leather purse	2.43	0.23	6.15	0.16	3.72	Experience

Mean values on a 7-point scale, where 1 indicates “not at all” and 7 indicates “very well.”

**TABLE 4:** 2 × 2 between-respondents factorial design.

		Product type		
		Search product (SP)	Experience product (EP)	Total
OPR type	System generated recommendation (SGR)	SGR × SP (126)	SGR × EP (113)	239
	Consumer generated recommendation (CGR)	CGR × SP (99)	CGR × EP (144)	243
Total		225	257	482

**TABLE 5:** Loadings and cross-loadings measures.

Items	ENJ	PDQ	PDE	RUI
IRU1	0.197	0.366	–0.157	**0.865**
IRU2	0.222	0.341	–0.149	**0.863**
IRU3	0.197	0.360	–0.163	**0.877**
IRU4	0.202	0.344	–0.148	**0.871**
PDE1	–0.078	–0.179	**0.851**	–0.193
PDE2	–0.056	–0.147	**0.916**	–0.132
PDE3	–0.058	–0.113	**0.942**	–0.093
PDE4	–0.057	–0.120	**0.928**	–0.076
PDQ1	0.266	**0.767**	–0.200	0.358
PDQ2	0.269	**0.816**	–0.187	0.303
PDQ3	0.271	**0.843**	–0.176	0.242
PDQ4	0.196	**0.743**	–0.134	0.430
PDQ5	0.245	**0.794**	–0.132	0.388
ENJ1	**0.877**	0.224	–0.095	0.146
ENJ2	**0.896**	0.207	–0.094	0.131
ENJ3	**0.901**	0.217	–0.109	0.174
ENJ4	**0.865**	0.206	–0.013	0.183
ENJ5	0.881	0.148	–0.005	0.156

Extraction Method: Principal Component Analysis. Rotation Method: Varimax with Kaiser Normalization. Rotation converged in 5 iterations. Bold values are exploratory factor analysis.

**TABLE 6:** Descriptive statistics and reliability analysis.

Constructs	Mean	SD	Cronbach’s α	CR	AVE
Intention to reuse OPR	4.53	1.24	0.913	0.938	0.854
Intention to purchase	4.64	0.81			
Perceived decision effort	2.43	0.87	0.954	0.981	0.873
Perceived decision quality	3.48	0.86	0.948	0.952	0.841
Perceived enjoyment	3.97	1.08	0.924	0.961	0.872

**TABLE 7:** Assessment of discriminant validity and correlation.

Constructs	IRU	ITP	PDE	PDQ	ENJ	OPR type	Product type
Intention to reuse OPR (IRU)	**0.977**						
Intention to purchase (ITP)	0.681	**1.000**					
Perceived decision effort (PDE)	–0.471	–0.214	**0.934**				
Perceived decision quality (PDQ)	0.792	0.641	–0.501	**0.917**			
Perceived enjoyment (ENJ)	0.501	0.364	–0.314	0.571	**0.934**		
OPR type	–0.426	–0.415	0.112	–0.349	0.051	**1.000**	
Product type	0.175	0.091	–0.168	–0.247	0.319	0.136	**1.000**

Diagonal values are the square root of AVE and off-diagonal values are correlation. Bold values are exploratory factor analysis.

### Non-response bias analysis

This study also analyses the non-response bias by contrasting the first 50 and last 50 responses that should not be statistically significant. A paired *t*-test is conducted on all study constructs and the results indicate that there was no statistically significant (*p* > 0.05) differences in the means of these two groups [IRU (Mean_E_ = 3.98, Mean_L_ = 3.95, *t* = –0.758), PDE (Mean_E_ = 2.37, Mean_L_ = 2.53, *t* = 1.258), PDQ (Mean_E_ = 3.61, Mean_L_ = 3.48, *t* = –0.035), and ITP (Mean_E_ = 3.48, Mean_L_ = 3.38, *t* = –0.843)]. However, it can be inferred that those who have not responded to the survey would have the same perceptions of the study constructs as those who have responded, and the results are unlikely to be affected.

## Data analysis

### Product categorization

For examining the interaction impact between OPR type (SGR & CGR) and product type (search & experience), we classified the products into search and experience products in terms of pre-purchase performance veracity (Xia and Bechwati, 2008; Park and Lee, 2009). Using a 7-point Likert scale with end points from “1 for Not at all” to “7 for Very well,” data about the products bought over six months and the ability to evaluate the product performance before and after use were collected to determine which product belongs to which product category. We also did a one-way ANOVA for examining the difference between the before and after purchase scale. The ANOVA results [*F*(1,23) = 53.475, *p* < 0.000] indicated a significant difference between search and experience products. As depicted in Table 3, laptops, cell phones, digital cameras, home electronics, photographic equipment, motorcycle parts, toys, kitchen utensils, DVD players, printers, electronic accessories, network equipment, and eyeglasses are categorized as search products, given the relatively higher mean scores on the “before use” scale. In contrast, software, books/magazines, movies/music CDs, clothing, shoes, perfume, cosmetics, cleaning products, pet supplies, watch, and leather purse are viewed as experience products, given the low mean scores on the “before use” scale.

### Group assignment and control checks: Online product recommendation type vs. product type

This study follows a 2 × 2 between-respondents factorial design for examining the interaction effect between OPR type (SGR vs. CGR) and product type (search vs. experience) on customers’ decision beliefs. Descriptive statistics showed that out of 482 respondents, 239 and 243 respondents had consulted SGR and CGR, respectively. Furthermore, it is identified that out of 239 respondents, 126 and 113 respondents had used SGR for buying search products and experience products, respectively, and out of 243 respondents, 99 and 144 respondents had used CGR for buying search products and experience products, respectively. A 2 × 2 between-respondents factorial design is presented in Table 4.

This study also conducted a multivariate analysis of variance (MANOVA) test for confirming respondents’ assignment to the 2 × 2 factorial design. The MANOVA results showed no significant differences in terms of respondents’ characteristics, such as gender (*F* = 0.172, *p* = 0.516), occupation (*F* = 2.143, *p* = 0.155), online buying experience (*F* = 1.372, *p* = 0.412), OPR usage experience (*F* = 2.145, *p* = 0.167), and familiarity with Amazon (*F* = 0.145, *p* = 0.614). However, it is inferred that respondents’ characteristics are not the cause of changes in customers’ decision beliefs.

### Assessment of measurement model

We conducted an exploratory factor analysis (EFA) to explore the underlying dimensions and to demonstrate adequate reliability and construct validity (Hair et al., 2010). All the items were inserted together into SPSS and run factor analysis without rotation. The inspection of the correlation matrix shows that the majority of the coefficients are above 0.30. The Kaiser Meyer-Olkin value of 0.955 exceeded the recommended value of 0.60, and Bartlett’s test of sphericity (χ^2^ = 11254.088, *p* < 0.001) achieved a statistical significance, supporting the factorability of the correlation matrix. As presented in Table 5, EFA results reveal that the items are strongly loaded on the intended constructs with all above 0.7 and no cross-loadings higher than 0.388.

Construct reliability is assessed by computing Cronbach’s α, composite reliability (CR), and average variance extracted (AVE) for each construct. As presented in Table 6, values of Cronbach’s α, CR, and AVE for all constructs are greater than 0.850, confirming the construct reliability (Chin, 2010).

Further discriminant validity of the constructs is examined by comparing the results of square roots of AVEs with inter-construct correlations. As shown in Table 7, all square roots of AVEs of the study constructs are greater than their inter-construct correlations, providing evidence of construct discriminant validity. Similarly, we also examined the measurement models of SGR and CGR subsamples. The results of both measurement models satisfied the reliability and validity criteria (the results are omitted here due to brevity).

Beside classical approaches of Fornell-Larcker criterion and cross-loadings, this study also applies the latest technique of heterotrait-monotrait (HTMT) ratio to examine the discriminant validity. We run a bootstrapping routine in SmartPLS 3 in order to obtain the HTMT results. Henseler et al. (2015) recommended the cut-off points of 0.85 and 0.90 for establishing a discriminant validity between two reflective constructs, whereas HTMT of 0.85 is the most conservative criterion. If the HTMT ratio is less than 0.85, then the discriminant validity between the two constructs is established. The HTMT results are presented in Table 8, showing that all HTMT ratios are below 0.85, indicating that discriminant validity is unlikely to be an issue in this study. Based on the classical and HTMT approaches of discriminant validity, it is confirmed that the discriminant validity is established in this study.

**TABLE 8:** HTMT results of discriminant validity.

Constructs	IRU	ITP	PDE	PDQ	ENJ	OPR type	Product type
Intention to reuse OPR (IRU)							
Intention to purchase (ITP)	0.721						
Perceived decision effort (PDE)	–0.581	–0.421					
Perceived decision quality (PDQ)	0.843	0.784	–0.629				
Perceived enjoyment (ENJ)	0.612	0.517	–0.511	0.681			
OPR type	–0.617	–0.589	0.375	–0.469	0.187		
Product type	0.342	0.245	–0.328	–0.348	0.485	0.278	

Diagonal values are the square root of AVE and off-diagonal values are correlation.

### Hypotheses testing

**FIGURE 2:**
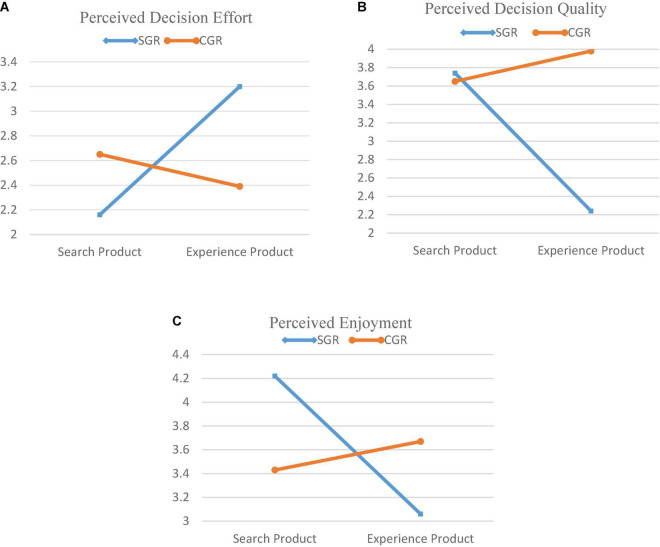
Interaction effect of OPR type and product type for perceived decision effort, perceived decision quality, and perceived enjoyment. **(A)** Interaction effect for PDE. **(B)** Interaction effect for PDQ. **(C)** Interaction effect for ENJ.

The significance of Bartlett’s test of sphericity suggested the suitability of multivariate analyses. We used two major data analysis techniques: MANOVA and partial lease square (PLS) for testing the study hypotheses and to generalize the findings. A multivariate test using MANOVA is conducted for measuring the interaction effect of OPR type (SGR vs. CGR) and product type (Search vs. Experience) on decision and affective assessment factors. The MANOVA results reveal a significant interaction effect (Pillai’s trace = 0.166, Wilks’λ = 0.834, Hotelling’s trace = 0.200, Roy’s largest root = 0.200, *F* = 31.819, *p* < 0.001). Table 9 summarizes the results of MANOVA, indicating a significant interaction effect between OPR type (SGR vs. CGR) and product type (search vs. experience) on perceived decision effort, perceived decision quality, and perceived enjoyment. Interaction effects are further examined through a simple effect analysis (Table 10) and graphical representation (Figure 2). The simple effect test is the follow-up of a statistical test when the interaction effect is significant. It helps explore the nature of the interaction effects by examining the difference between groups within one level of one of the independent variables. The graphical representation of the interaction effect also gives a quick illustration of the pattern of the simple effect results.

**TABLE 9:** MANOVA results.

	Interaction effect OPR type*Product type				

**Dependent variables**	**Sum of squares**	**df**	**Mean square**	***F***	**Sig.** **(*P*-value)**
Perceived decision effort	21.206	1	21.206	30.002	0.000
Perceived decision quality	31.141	1	31.141	36.405	0.000
Perceived enjoyment	51.534	1	51.534	75.118	0.000

**TABLE 10:** Simple effect analysis.

		OPR type				
Variables	Product type	SGR (I)	CGR (J)	Mean difference (I-J)	*F*	Significance
Perceived decision effort (DE)	Search	2.16	2.65	-0.49	15.168	0.000
	Experience	3.20	2.39	0.81	48.818	0.000
Perceived decision quality (DQ)	Search	3.76	3.65	0.11	9.878	0.000
	Experience	2.24	3.98	-1.74	251.219	0.000
Perceived enjoyment (PE)	Search	4.22	3.43	0.79	39.509	0.000
	Experience	3.06	3.67	-0.61	28.589	0.000

## Perceived decision effort

As the interaction effect for a perceived decision effort is significant (*F* = 30.002, *p* < 0.001), the simple effect analysis further shows that the customer perception significantly lower decision effort with SGR (compared to CGR) for shopping search products than for experience products (M*_differenc_*_E_ = –0.49, *F* = 15.168, *p* < 0.001), whereas customer perception significantly lower decision effort with CGR (compared to SGR) for shopping experience products than search products (M*_difference_* = 0.81, *F* = 48.818, *p* < 0.001). More closely, both mean differences indicate that customers perceived comparatively lower decision effort for shopping search products based on SGR (M*_difference_* = –0.49) than shopping experience products based on CGR (M*_difference_* = 0.81). Hence, the customer perceived differences in decision effort while using SGR and CGR for buying different types of products. Further as depicted in Figure 2A, the mean values of perceived decision effort are plotted for the interaction effect between OPR type and product type. The result endorses the above findings which show that the customer perceived lower decision effort with SGR (compared to CGR) for search products. Conversely, when moving from search product to experience product, perceived decision effort increases while using SGR and decreases while using CGR which further endorses the above findings of the simple effect analysis. Thus, H1 is empirically supported.

### Perceived decision quality

As the interaction effect for a perceived decision quality is significant (*F* = 36.405, *p* < 0.001), the simple effect analysis further shows that the customer perceived significantly greater decision quality with SGR (compared to CGR) for search products than for experience products (M*_differen*ce*_* = 0.11, *F* = 9.878, *p* < 0.001), whereas the customer perceived significantly greater decision quality with CGR (compared to SGR) for shopping experience products than search products (M*_difference_* = –1.74, *F* = 251.219, *p* < 0.001). More closely, the mean difference in decision quality of OPR use between SGR and CGR is greater for experience products (M*_difference_* = –1.74) than for search products (M*_difference_* = 0.11), indicating that the CGR is most suitable in terms of decision quality for shopping experience products than search products. Hence, the customer perceived differences in decision quality in using SGR and CGR for buying different types of products. Further as presented in Figure 2B, the mean values of perceived decision quality are plotted for the interaction effect between OPR type and product type, indicating that consumers perceived higher decision quality with SGR (compared to CGR) for search products. Conversely, when moving from search product to experience product, perceived decision quality increases while using CGR and largely decreases while using SGR that further endorses the above findings of the simple effect analysis. Thus, H2 is empirically supported.

### Perceived enjoyment

As the interaction effect for perceived enjoyment is significant (*F* = 75.118, *p* < 0.001), the simple effect analysis further reveals that customers perceived significantly greater enjoyment with SGR (compared to CGR) for shopping search products than experience products (M*_difference_* = 0.78, *F* = 39.509, *p* < 0.001). In contrast, consumers perceived significantly greater enjoyment with CGR (compared to SGR) for shopping experience products than for search products (M*_difference_* = –0.61, *F* = 28.589, *p* < 0.001). Hence, customers perceived differences in enjoyment with SGR and CGR for buying different types of products. Furthermore, as depicted in Figure 2C, the mean values of perceived enjoyment are plotted for the interaction effect, indicating that customers perceived greater enjoyment with SGR (compared to CGR) while shopping search products. Conversely, when moving from search product to experience product, perceived enjoyment decreases while using SGR and increases while using CGR for shopping experience products which further endorses the above findings of the simple effect analysis. Thus, H3 is empirically supported.

### Outcome measure

**FIGURE 3:**
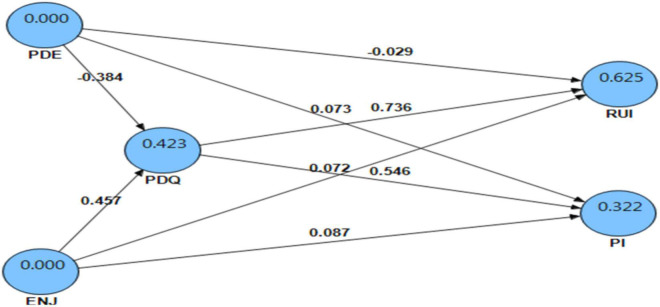
Partial lease square results.

Analysis of PLS using SmartPLS 2.0 is conducted to examine the following hypotheses: H4a, H4b, H5a, H5b, H6a, and H6b based on the relationship between process measures and outcome measures, intention to reuse OPR, and intention to purchase (see Figure 3). Perceived decision effort (*b* = –0.029, *t* = 3.014, *p* < 0.01), perceived decision quality (*b* = 0.736, *t* = 86.947, *p* < 0.001), and perceived enjoyment (*b* = 0.546, *t* = 6.269, *p* < 0.001) significantly affect customer’s intention to reuse OPR (R^2^ = 0.625). Similarly, perceived decision effort (*b* = –0.073, *t* = 6.612, *p* < 0.001), perceived decision quality (*b* = 0.072, *t* = 3.156, *p* < 0.01), and perceived enjoyment (*b* = 0.087, *t* = 6.612, *p* < 0.001) significantly influence customer’s intention to purchase (*R*^2^ = 0.322). Therefore, H4a, H4b, H5a, H5b, H6a, and H6b are supported.

### Supplementary analysis

In line with the effort-accuracy model, the fit between OPR type and product type leads to lower decision effort and higher enjoyment, which could improve the perceived OPR decision quality. As shown in Figure 3, results from PLS further reveal a significant impact of perceived decision effort (*b* = –0.384, *t* = 26.901, *p* < 0.001) and perceived enjoyment (*b* = 0.457, *t* = 31.932, *p* < 0.001) on consumer’s perceived decision quality (R^2^ = 0.423). However, customers’ lower decision effort and greater enjoyment with the OPR significantly enhance their buying decision quality.

## Discussion

This study improves our understanding of how consumers evaluate the OPR and the conditions under which consumers perceive greater decision and affective assessment of the focal recommendation (i.e., SGR or CGR). The results suggest that the effect of OPR use on customers’ decisions and affective assessment of the focal recommendation depends on the product type. Under matching conditions, consumers perceive relatively lower decision effort, greater decision quality, and higher enjoyment with OPR use. Moreover, the results indicate that more OPR usefulness is perceived in terms of decision quality, enjoyment, and lower decision effort, thus consumers express more intentions to purchase and reuse OPR. The results also reveal that perceived decision effort and perceived enjoyment with the OPR significantly influence customers’ buying decision quality.

Additionally, consumers perceive greater evaluation ability of SGR than CGR for shopping search products than experience products and they perceive greater evaluation ability of CGR (compared to SGR) for shopping experience products (compared to search products). Specifically, the results indicate that consumers’ decision and affective assessments are superior with CGR (compared to SGR) for shopping experience products than search products. Conversely, all these beliefs are superior in SGR (compared to CGR) for shopping search products than experience products. It is due to the similarity between SGR and search products and between CGR and experience products in terms of information required for product evaluation [i.e., attribute-based products require attribute-based information (i.e., SGR), experience-based products require experience-based information (i.e., CGR)] because different types of products require related information (Mudambi and Schuff, 2010; Ashraf et al., 2017). Thus, all identified relationships regarding the interaction effects on consumers’ evaluation beliefs indicate the recommendation-product congruence in terms of their effect mechanisms, which provide empirical support to our hypothesized relationships developed based on CFT and SCT.

Our findings under matching conditions indicate that attribute-based recommendation (i.e., SGR) describing search products and experience-based recommendation (i.e., CGR) explaining experience products leads the consumers to perceive lower decision effort, greater decision quality, and higher enjoyment with the use of focal recommendation. In contrast, attribute-based recommendation (i.e., SGR) describing experience products and experience-based recommendation (i.e., CGR) explaining search products lead the consumers to perceive greater decision effort, lower decision quality, and less enjoyment while using the OPR for making a purchase. Hence, the results are consistent with the underlying reasoning of CFT that if both problem representation and problem-solving tasks emphasize the same information, then a cognitive fit is attained which subsequently leads to the development of a consistent mental representation. Therefore, this consistent mental representation or a cognitive fit leads to developing recommendation-product congruence. Otherwise, the inconsistent mental representation causes incongruity and unfavorable perception of OPR evaluation.

Schema congruity theory complements the CFT for explaining the cognitive mechanisms underneath the representation of a problem that would fit the problem-solving task to develop a consistent mental representation. The processing of OPR (SGR or CGR) is influenced by two schemas: brain-stored and stimulus-based schema. The brain-stored schema (i.e., a piece of product information stored in the brain) that matches the schema of incoming information (i.e., stocktickerSGR and CGR) enables consumers to formulate a consistent mental representation and assessment of the product. Particularly, customers’ intention of searching for information on different products may trigger their different schemas of the brain. For example, customers’ brain-stored schemas for different products guide their behavior for subsequent acquisition and processing of the product-related information. Searching information on an experienced product activates a holistic schema of the brain, whereas, a search product activates an analytic schema of the brain. The customer’s response to the incoming information represented by a particular schema relies on the congruity of the schema with the existing brain-stored schema. The customer follows a schema-based information-processing strategy based on the congruity between the stimulus-based schema of OPR and the existing brain-stored schema. Consequently, it leads customers to formulate a consistent mental representation that helps to process the information with superior evaluation beliefs. For instance, the brain-stored schema of evaluating a smartphone renders a judgment based on its key features, such as the battery, screen, and memory size. When this incoming information is closely matched with the existing related schema, then schema congruity occurs, which helps the customers to process the information or to evaluate the product with minimal cognitive elaboration and greater perception of OPR usefulness. In contrast, if a mismatch between stimulus- and brain-stored schemas occurs, then the schema incongruity leads the customers to adopt a piecemeal strategy of information processing and exerts a greater effort in integrating the two schemas. Based on the SCT, a customer’s search for a focal product type activates his or her brain-stored schema. Depending on the customer’s intention to buy a search product or an experienced product, a brain-stored schema representation of attribute- or experience-based information is initiated and established, respectively. Therefore, a customer uses schema-based information evaluation strategy when OPR type (i.e., stimuli-based-schema of SGR or CGR) fits the schema of the brain triggered by search or experience products, resulting in a consistent mental representation which subsequently enhances the perceptions of consumers’ decision and affective assessment of the focal recommendation (i.e., SGR or CGR). There could be the following possible explanations.

First, consumers would conceive a recommendation to be more useful and related to their product judgment when the recommendation (SGR or CGR) matches the product type (search or experience). It is because the degree of information relevance causes the consumer’s involvement, which would enhance the motivation for processing information more elaborately (Petty and Cacioppo, 1986; Huang et al., 2013). The effective processing of recommendations under matching conditions enables the consumers to better evaluate the product, which in turn leads to a better perception of OPR assessment factors.

Second, consumers will be involved in a deeper evaluation of the recommendation when it matches their existing knowledge structure activated by the buying task of the focal product. Consequently, the consumers’ involvement in deeper recommendation evaluation leads the consumers to have a better decision and affective assessment of the recommendation.

Third, prior information processing studies (e.g., Lee and Labroo, 2004) reported that individuals’ cognitive effort in processing information is closely related to the pattern and fluency of the information. In processing a recommendation under the matching condition, a consumer would fluently process the recommendation that is presented in a consistent pattern. However, a consumer feels lower decision effort in processing the recommendation for product evaluation as he or she is more fluent under matching conditions due to his or her consistent mental representation. Therefore, the results support our argument that when the OPR type matches the product type, it would lead to perceiving lower decision effort to process the recommendation.

Fourth, under a mismatching condition, a greater cognitive effort required to process information causes negative consumer sentiments, such as dissatisfaction, and subsequently, it diminishes consumers’ motivation to process the information (Meyers-Levy and Tybout, 1989; Garbarino and Edell, 1997). Therefore, if the OPR type matches the product type, consumers’ negative sentiments generated from such a mismatch may cause them to adopt an indifferent attitude toward processing the OPR. In conclusion, the results strengthen our theoretical explanation that product-recommendation congruity would lead to the consumers’ better decision and affective assessment of the OPR.

## Research implications

This study provides a number of significant contributions to the literature and practice. First, this study proposes and empirically tests the recommendation-product congruity proposition. Our results reconcile with the prior contradicting findings regarding the differential impacts of attribute- and experience-based information on consumers’ evaluation beliefs. For example, past studies (Park and Lee, 2009; Benlian et al., 2012; Huang et al., 2013) claim that attribute-based recommendations are more useful than experience-based ones as it examines tangible product attributes. Contrarily, other studies (Bei et al., 2004; Franke et al., 2004) found that compared to attribute-related information, experience-related information is more valuable because it assists potential customers in better visualizing and understanding the use of the product.

Past studies based on the effort-accuracy model (e.g., Häubl and Trifts, 2000; Häubl and Murray, 2003; Häubl et al., 2004; Fasolo et al., 2005; Hostler et al., 2005; Xiao and Benbasat, 2007; Xu et al., 2014) also reported mixed findings regarding the trade-off between decision effort and accuracy for using recommendations. For example, Fasolo et al. (2005) demonstrated that experience-based information (i.e., SGR) not only enhances the decision quality but also increases the decision effort. Benbasat and Todd (1996) reported that customers use attribute-based information (i.e., SGR) to conserve cognitive effort, not certainly to increase their decision quality (accuracy). Our study reconciled with the diverse findings by identifying the OPR type (SGR & CGR) that is perceived to be useful (in terms of greater decision and affective assessment of the OPR) when emphasizing a particular product type (search & experience) based on the CFT which is supplemented by the SCT with a possible impact of the product type (Mudambi and Schuff, 2010). Under a matching condition (SGR for search product and CGR for experience product), such a recommendation is considered superior in terms of greater decision quality and enjoyment while lower cognitive effort is required from consumers for the OPR use. Under a mismatching condition, the consumers perceive greater difficulty to evaluate the recommendation, which results in negative influence on the OPR assessment beliefs.

Second, while most of the prior studies applied the CFT to examine the direct effects of antecedents on outcomes (e.g., Hong et al., 2004), least attention has been paid to investigating the internal mechanisms of how the OPR content matching with the product type could lead to formulating a consistent mental representation for generating a favorable decision and affective assessment of OPR. We used the SCT in complement with the CFT as an underlying theoretical underpinning for examining and explaining the “black box” (internal mechanism) of how the congruity between recommendation and product leads the consumers to perceive favorable recommendation assessment by formulating a consistent mental representation.

Third, while past studies (e.g., Ashraf et al., 2019, 2020) emphasized on the effect of evaluation beliefs on behavioral intention, less focus has been placed to explore the antecedents of consumers’ beliefs of OPR assessment. This study extends the prior findings by empirically testing the interaction impact between the OPR type and the product type as antecedents on consumers’ decisions and affective assessment of OPR use.

Fourth, this study also provides practical implications to Web designers and developers who are interested to integrate SGR and CGR mechanisms for evaluating different products. The web developers can integrate SGR and CGR into the product recommendation algorithm for generating the recommendations. In other words, the web developers can find an approach that considers the product type which is congruent with the OPR type for positioning the SGR or the CGR ahead of other types of recommendations. Employing such a technique will enable consumers for making the right product judgment and buying decisions. The importance of the approach relies on the fact that under the matching condition, consumers perceive the OPR (SGR or CGR) to be more valuable for making a quality decision with lower cognitive effort. Nevertheless, distinguishing the OPR type and generating the recommendation relevant to the product type interest to the consumers remains a challenge. Future studies could apply text-mining techniques for analyzing the OPR contents and presenting them to consumers for product types. It requires the administration of the web to provide a distinct definition of SGR and CGR as either attribute- or experience-based.

## Limitations and future directions

First, this study considered only SGR and CGR related to attribute-based information and experience-based information, respectively. The significant differences existing between these two types of recommendations (SGR & CGR) have been reported to have a significant differential impact on consumer perceptions of the recommendations (Benlian et al., 2012; Ashraf et al., 2017, 2019). Nevertheless, future studies can further explore an ideal combination of attribute- and experience-based recommendations that could improve consumers’ decisions and affective assessment of the recommendations. Second, we could not directly measure consumers’ schema of acquiring information because it is a theoretical concept that is difficult to grasp; alternatively, the survey method could be used (Huang et al., 2013). We expected that consumers would consult SGR as they intend to buy a search product (i.e., cell phone) and would refer to CGR as they intend to buy an experienced product (i.e., cloth). Although we did not determine fully that all the respondents activated information acquisition schema for different types of products, we tried to ascertain it by establishing 2 × 2 factorial designs based on the data collected through the survey method in the natural setting of Amazon. Future studies may further explore this phenomenon in an experimental setting. Third, we used a cross-sectional survey method rather than the longitudinal method for collecting the data. Future studies could employ a longitudinal research method for exploring temporal and causal relationships over a particular time frame. Fourth, the study is conducted in the context of Amazon which is a popular e-commerce platform. Future studies can consider less popular e-commerce platforms for generalizing our findings. Fifth, we consider only search and experience products. Future studies could consider other types of products, such as physical vs. digital products or high- vs. low-involvement products. Finally, future studies could also use the CFT and SCT to examine conventional information system adoption by testing the similar nature of the congruity proposition.

## Conclusion

This study empirically tested the recommendation-product congruity proposition by examining the interaction effect of recommendation type (SGR vs. CGR) and product type (search vs. experience) on consumers’ decision and affective assessment behavior of the focal recommendation. We empirically tested to find if consumers’ decisions and affective assessment of the OPR are significantly affected under the matching condition between OPR type and product type. By integrating the CFT and the SCT, we explained how the congruity between the recommendation and the product leads the consumers to perceive a favorable assessment of the OPR. When the recommendation matches the product described, such as SGR describing a search product and CGR describing an experienced product, consumers perceive the recommendation as more valuable in terms of lower decision effort, greater decision quality, and enjoyment of their product judgment. Additionally, consumers express more intentions to purchase and reuse OPR, when they perceive greater decision and affective assessment of the recommendation. The empirically tested recommendation-product congruity proposition guides the provision of OPR (SGR vs. CGR) in online shopping platforms.

## Data availability statement

The raw data supporting the conclusions of this article will be made available by the authors, without undue reservation.

## Author contributions

YL and MA contributed to conceptualizing, designing, analyzing, and writing up the research. O and WS handled data collection, curation, and data visualization. JA and MK conducted literature review and hypotheses development. MK contributed in result discussion and implications. MA wrote the original draft and handled project administration. All authors contributed to the article and approved the submitted version.
